# Synergistic antiallodynic effects of pregabalin and thioctic acid in a rat model of neuropathic pain

**DOI:** 10.3389/fphar.2025.1675015

**Published:** 2025-09-01

**Authors:** Edith Zárate, Vinicio Granados-Soto, Oscar Arias-Carrión

**Affiliations:** ^1^ Psicofarma S.A. de C.V., Mexico City, Mexico; ^2^ Neurobiology of Pain Laboratory, Departamento de Farmacobiología, Cinvestav, South Campus, Mexico City, Mexico; ^3^ División de Neurociencias Clínica, Instituto Nacional de Rehabilitación Luis Guillermo Ibarra Ibarra, Mexico City, Mexico; ^4^ Tecnologico de Monterrey, Escuela de Medicina y Ciencias de la Salud, Mexico City, Mexico

**Keywords:** neuropathic pain, pregabalin, thioctic acid, isobolographic analysis, polypharmacy, calcium channels, oxidative stress, fixed-dose formulation

## Abstract

**Background:**

Neuropathic pain is a chronic and often disabling condition that remains refractory to monotherapy because of limited efficacy and dose-limiting adverse effects. Combination therapies that engage complementary mechanisms of action offer a rational strategy to enhance efficacy while minimizing toxicity. Pregabalin, a ligand of α_2_δ subunits of voltage-gated calcium channels, reduces presynaptic calcium influx and glutamate release. In contrast, thioctic acid (α-lipoic acid), a potent antioxidant with anti-inflammatory properties, modulates TRPV1 channel expression and activity and inhibits CaV3.2 T-type calcium channels. Both agents exert antiallodynic effects in preclinical models, yet their pharmacodynamic interaction has not been systematically evaluated. Given their distinct but convergent actions on neuronal excitability and pain signalling, quantitative synergy analysis is warranted to define the therapeutic potential of their combined use.

**Methods:**

We evaluated the antiallodynic efficacy of pregabalin and thioctic acid, alone and in combination, in female Wistar rats subjected to L5–L6 spinal nerve ligation. Mechanical withdrawal thresholds were measured using von Frey filaments up to 8 h post-oral administration. Dose–response curves were generated for each monotherapy and its 1:1 fixed-ratio combination. Isobolographic analysis was conducted to quantify pharmacodynamic interactions. All behavioural testing was performed under blinded conditions, and adverse effects were qualitatively monitored.

**Results:**

Both compounds produced dose-dependent increases in mechanical withdrawal thresholds (antiallodynic effects), with ED_50_ values of 2.45 ± 0.23 mg/kg for pregabalin and 57.49 ± 5.59 mg/kg for thioctic acid. The 1:1 fixed-ratio combination yielded an ED_50_ of 15.7 ± 1.0 mg/kg and a maximal %MPE of 72.3% ± 4.8%. Isobolographic analysis demonstrated a synergistic interaction, with an interaction index (γ) of 0.524 (95% CI: 0.41–0.66; p < 0.05 vs. theoretical ED_50_). No overt adverse effects were observed at combination doses, whereas mild sedation occurred only at the highest pregabalin monotherapy dose.

**Conclusion:**

These findings provide robust preclinical evidence that co-administration of pregabalin and thioctic acid produces synergistic antiallodynic effects in a validated model of neuropathic pain. This interaction enables effective analgesia at reduced doses, supporting a potential tolerability advantage. Our data support further investigation of this combination in chronic dosing paradigms, inclusion of both sexes, and clinical translation.

## 1 Introduction

Neuropathic pain is a chronic and debilitating condition that often persists well beyond the resolution of the initiating injury. Defined by the International Association for the Study of Pain as pain arising from a lesion or disease of the somatosensory system, it is characterized by spontaneous sensations such as burning, tingling, or electric shocks, as well as stimulus-evoked responses including mechanical allodynia and hyperalgesia (Geber et al., 2009; Wang et al., 2013; Descalzi et al., 2015). These symptoms disrupt sleep, impair mood, and compromise daily functioning, with significant personal and socioeconomic consequences. Despite its high prevalence, neuropathic pain remains difficult to manage with currently approved pharmacological treatments (Kaye et al., 2025). Sex-dependent differences in pain processing and drug responsiveness are well documented (Mogil, 2020), yet most preclinical synergy studies have been conducted exclusively in male rodents. This underlines the need for research in underrepresented populations, including females, to better inform translational relevance.

First-line therapies—including tricyclic antidepressants, serotonin–norepinephrine reuptake inhibitors, and calcium channel modulators—offer only partial relief in many patients and are often limited by dose-dependent adverse effects such as sedation, dizziness, gastrointestinal discomfort, and cognitive impairment (Dworkin et al., 2007; Baron, 2009; Attal, 2012). These limitations have prompted growing interest in mechanism-based polypharmacy strategies, in which agents with complementary pharmacological profiles are combined to enhance efficacy while mitigating side effects. Such combinations may also enable clinically meaningful dose reductions, improving tolerability without compromising analgesic benefit, a strategy supported by recent isobolographic synergy studies in neuropathic pain models (Zamora-Diaz et al., 2025).

Pregabalin, a structural analogue of γ-aminobutyric acid, is a selective ligand of the α_2_δ-1 subunit of voltage-gated calcium channels. This drug inhibits calcium influx at presynaptic terminals, reduces glutamate release and suppresses excitatory neurotransmission within pain pathways. In addition to its canonical calcium channel–modulating action, pregabalin has been shown to influence other molecular targets, including excitatory amino acid transporters, potassium channels, and purinergic receptors (Yu et al., 2013; Verma et al., 2014). Its analgesic efficacy in neuropathic pain has been demonstrated in both animal models and clinical studies; however, its therapeutic index is often restricted by central nervous system effects at higher doses (Zaccara et al., 2011; Derry et al., 2019; Mayoral et al., 2024).

Thioctic acid (α-lipoic acid), an endogenously synthesized disulfide compound, possesses potent antioxidant and anti-inflammatory properties. It is clinically approved for the treatment of diabetic polyneuropathy and acts by scavenging reactive oxygen species, modulating redox-sensitive transcription factors, and downregulating pro-inflammatory cytokines (Ziegler et al., 2006; Mijnhout et al., 2012; Papanas and Ziegler, 2014; Lazutka et al., 2024). In preclinical models, thioctic acid has demonstrated antiallodynic effects across multiple forms of nerve injury, including vincristine-induced neuropathy, spinal nerve ligation, and ischemia–reperfusion injury (Mitsui et al., 1999; Memeo and Loiero, 2008; Kahng et al., 2015). Notably, thioctic acid also inhibits T-type Ca_V_3.2 channels, a class of voltage-gated calcium channels implicated in peripheral sensitization and the amplification of nociceptive signals, and modulates TRPV1 channels by reducing their activity and expression through antioxidant and NF-κB–dependent mechanisms (Lee et al., 2009; Zhang et al., 2020; Yazğan et al., 2023). Its distinct mechanism and favourable safety profile make it an attractive candidate for combination strategies despite not being a first-line analgesic.

Given their distinct yet potentially complementary mechanisms—central calcium channel inhibition via α_2_δ modulation by pregabalin and peripheral antioxidant and ion channel–regulatory effects mediated by thioctic acid—the combination represents a rational therapeutic strategy. Prior studies have yielded mixed results: preclinical work suggested interaction-dependent analgesic enhancement (Cruz-Alvarez et al., 2018), while clinical trials have reported either synergistic (Park et al., 2022) or neutral (Gilron et al., 2024) outcomes. These discrepancies highlight the need for a controlled, quantitative assessment of their pharmacodynamic interaction. Potential pharmacokinetic interactions between pregabalin and thioctic acid are expected to be minimal due to their distinct elimination pathways—renal excretion unchanged for pregabalin and hepatic β-oxidation for thioctic acid (Shay et al., 2009; Schulze-Bonhage, 2013)—although effects on gastrointestinal absorption or transporter modulation cannot be excluded.

In this study, we employed the isobolographic analysis to determine whether the combined administration of pregabalin and thioctic acid produces additive, synergistic, or antagonistic effects in a rat model of L5–L6 spinal nerve ligation. To address the literature gap, experiments were performed exclusively in female rats, providing novel synergy data in an underrepresented sex. This approach aims to establish a mechanistic and formulation-based foundation for the development of dose-efficient combination therapies in the treatment of neuropathic pain.

## 2 Materials and methods

### 2.1 Animals

Female Wistar rats (120–140 g) were used in all experiments. The exclusive use of females was deliberate, to address the persistent sex imbalance in preclinical neuropathic pain research, where most synergy studies have been performed in males (Mogil, 2020). While this limits generalisability, it provides novel pharmacological synergy data in an underrepresented sex. Animals were housed in groups of three under standard laboratory conditions (22 ± 1 °C, 12-h light/dark cycle) with unrestricted access to food and water. All procedures conformed to the ethical guidelines of the International Association for the Study of Pain (Zimmermann, 1983), the Mexican NOM-062-ZOO-1999 regulation, and the NIH Guide for the Care and Use of Laboratory Animals. The study protocol (B13-16) was reviewed and approved by both the Research Committee (INER/CI/125/16) and the Research Ethics Committee (INER/CEI/173/16) of the Instituto Nacional de Enfermedades Respiratorias Ismael Cosío Villegas, with validity from June 2016 to June 2018. Each rat was used in a single experimental condition and euthanized by CO_2_ inhalation immediately following the final behavioural assessment. Animals exhibiting motor deficits after surgery were excluded. Sample sizes were determined to ensure statistical power while minimizing the use of animals. All behavioural assessments were performed under blinded conditions, with experimenters unaware of treatment allocation. The synergistic combination of pregabalin and α-lipoic acid for neuropathic pain is protected under patent family MX392835B (Mexico), US12029727B2 (USA), and CA3047077C (Canada); EP3593795A4 (Europe) remains under examination, with coverage valid at least until December 16, 2036.

### 2.2 Drugs and formulations

Pregabalin ((S)-3-isobutyl-GABA) and thioctic acid (a-lipoic acid) were suspended in 0.9% saline containing 0.5% carboxymethylcellulose and administered orally (p.o.) at freshly prepared doses. Combination doses (pregabalin 0.15–1.2 mg/kg + thioctic acid 3.6–28.8 mg/kg) were selected using the Tallarida equieffective ratio method, based on monotherapy ED_50_ values from preliminary experiments and literature (Kahng et al., 2015; Cruz-Alvarez et al., 2018), ensuring proportional contribution from each agent while remaining below doses associated with monotherapy adverse effects. Treatments were evaluated both as co-administered suspensions in a fixed 1:1 ratio (ED_50_-based) and as co-administered suspensions in a fixed 1:1 ratio (ED_50_-based). Anaesthesia for surgical procedures was induced via intraperitoneal injection of ketamine (45 mg/kg) and xylazine (12 mg/kg) (Sigma-Aldrich, St. Louis, MO).

### 2.3 Neuropathic pain induction

Neuropathic pain was induced via L5–L6 spinal nerve ligation following the method of Kim and Chung (1992). A left paraspinal incision was made to expose the L5 and L6 spinal nerves, which were tightly ligated with 6–0 silk suture. Sham controls underwent identical surgical procedures without ligation. Animals were allowed to recover for 14 days postoperatively. All behavioural experiments were conducted during the light phase.

### 2.4 Behavioural assessment of mechanical allodynia

Mechanical sensitivity was measured using calibrated von Frey filaments (0.4–15 g), applied to the plantar surface of the left hind paw through a wire mesh floor as described by Chaplan et al. (1994). Rats were acclimated for 30 min prior to testing. Withdrawal thresholds were calculated using the up–down method, starting at 2 g. Baseline values were recorded prior to drug administration. Post-treatment thresholds were assessed at 0.5, 1, 2, 3, 4, 5, 6, 7, and 8 h. The cutoff value for response was set at 15 g. An increase in withdrawal threshold was interpreted as an antiallodynic effect. Cold allodynia and deep tissue/mechanical hyperalgesia were not evaluated in this study to maintain methodological focus on mechanical allodynia as the primary outcome; this is acknowledged as a limitation and an avenue for future multimodal assessment.

### 2.5 Dose–response and %MPE calculations

Dose–response curves for pregabalin (0.3–30 mg/kg), thioctic acid (10–300 mg/kg), and their combination (3.75–30 mg/kg, 1:1 ratio) were generated by calculating the per cent maximal possible effect (%MPE) using the formula:

$$\%MPE=\frac{{AUC}_{Post-drug}-{AUC}_{Vehicle}}{{AUC}_{Sham}-{AUC}_{Vehicle}}\times 100$$

Sham-operated animals served as the 100% reference, while vehicle-treated ligated rats defined the 0% baseline. Experimental ED_50_ values were derived from non-linear regression of the dose–response data (Figure 2), and the results are summarised in Table 1.

**TABLE 1:** Antiallodynic efficacy and ED_50_ values of pregabalin, thioctic acid, and their combination.

Treatment	Maximal efficacy (%MPE, mean ± s.e.m.)	ED_50_ (mg/kg, mean ± s.e.m.)	Goodness of fit (*R* ^2^)
Pregabalin	108.2 ± 1.6	2.45 ± 0.23	0.995
Thioctic acid	91.3 ± 1.8	57.49 ± 5.59	0.890
Pregabalin + Thioctic acid (1:1)	72.3 ± 4.8	15.7 ± 1.0 (experimental)	0.989

*ED_50_ values were calculated from dose–response curves for %MPE (maximal possible effect) shown in Figure 2. — indicates values not calculated for fixed-dose capsules.

### 2.6 Isobolographic analysis of drug interaction

To assess synergy, isobolographic analysis was conducted by comparing the experimentally determined ED_50_ of the fixed-ratio combination with the theoretical additive ED_50_, calculated using the Tallarida method (Tallarida, 2000). The interaction index (γ) was computed as:

$$\gamma =\frac{{ED}_{50}^{experimental}}{{ED}_{50}^{theoretical}}$$

A value of γ < 1 indicates synergism, γ = 1 denotes additivity, and γ > 1 suggests antagonism. Experimental and theoretical ED_50_ values were statistically compared using Student’s t-test. Confidence intervals (95%) were determined for γ (Figure 3; Table 2).

**TABLE 2:** Isobolographic analysis of the pregabalin–thioctic acid combination.

Parameter	Value (mean ± s.e.m. or range)	Interpretation
Experimental ED_50_ (mg/kg)	15.7 ± 1.0 (95% CI: 13.9–17.5)	Lower than the theoretical ED_50_
Theoretical ED_50_ (mg/kg)	30.0 ± 2.8 (95% CI: 26.1–33.9)	Calculated from monotherapy data
Interaction index (γ)	0.524 (95% CI: 0.41–0.66)	Synergistic interaction (γ < 1)
Student’s t-test (Exp. vs. Theor. ED_50_)	p < 0.05	Statistically significant

**FIGURE 1:**
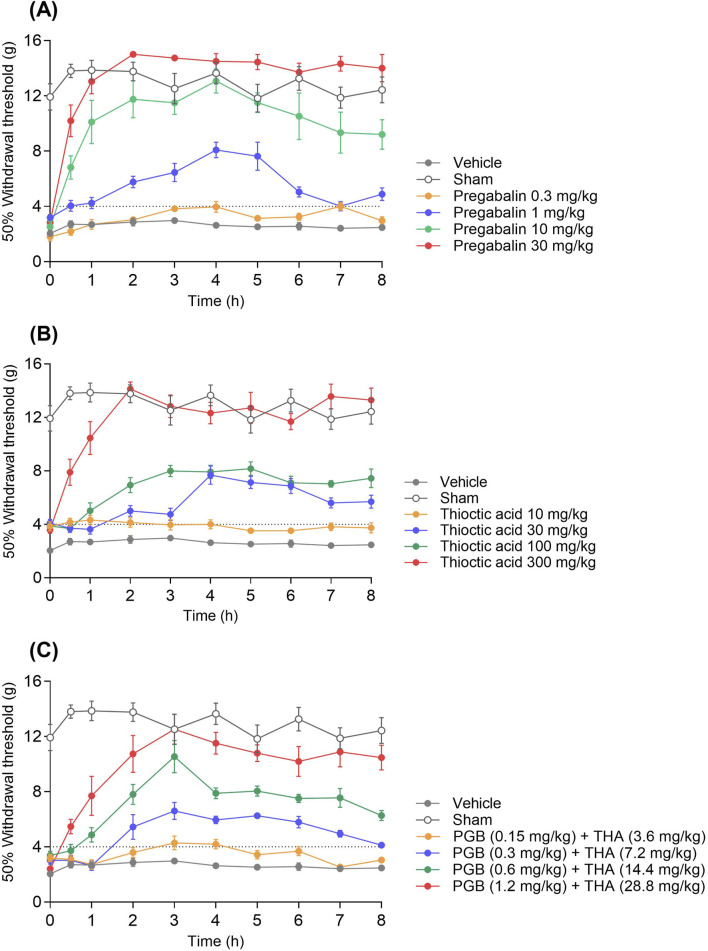
Dose-dependent effects of pregabalin, thioctic acid, and their combination on mechanical withdrawal thresholds in a neuropathic pain model. **(A)** Time course of mechanical withdrawal thresholds following administration of pregabalin (PGB) at 0.3, 1, 10, and 30 mg/kg. **(B)** Effects of thioctic acid (THA) at 10, 30, 100, and 300 mg/kg on withdrawal thresholds. **(C)** Combined administration of subtherapeutic doses of PGB (0.15–1.2 mg/kg) and THA (3.6–28.8 mg/kg) synergistically increased withdrawal thresholds over 8 h. In all panels, data are presented as mean ± standard error of the mean (s.e.m.). Withdrawal threshold was measured as the force (g) required to elicit a 50% paw withdrawal response. Sham and vehicle groups served as negative controls. The dotted line indicates the 4 g allodynia threshold.

**FIGURE 2:**
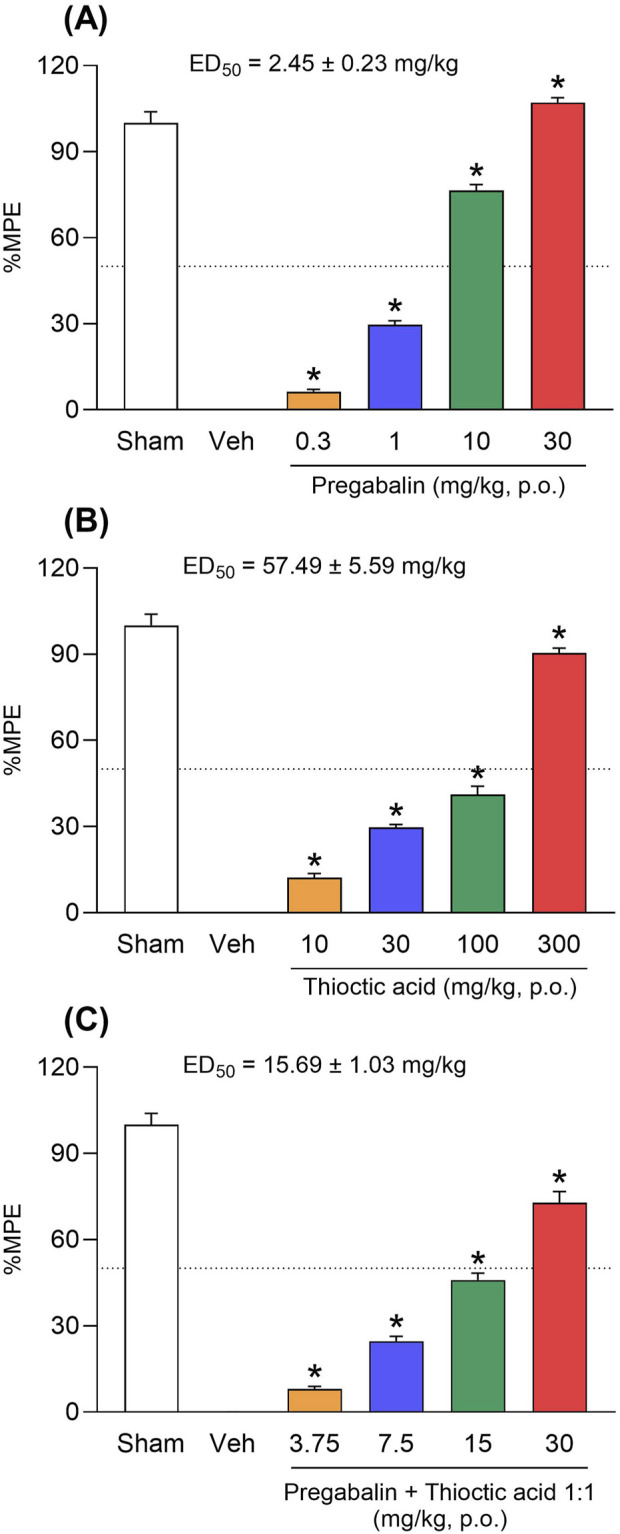
Antinociceptive efficacy and dose–response profiles of pregabalin, thioctic acid, and their fixed-dose combination. **(A)** Dose-dependent increase in maximal possible effect (%MPE) following oral administration of pregabalin (0.3–30 mg/kg) in a neuropathic pain model. **(B)** Thioctic acid produced a dose-dependent increase in %MPE at 10–300 mg/kg. **(C)** A fixed-dose combination of pregabalin and thioctic acid (in a 1:1 ratio, 3.75–30 mg/kg) produced enhanced antinociceptive effects. ED_50_ values (±s.e.m.) were calculated for each treatment: pregabalin, 2.45 ± 0.23 mg/kg; thioctic acid, 57.49 ± 5.59 mg/kg; combination, 15.69 ± 1.03 mg/kg. Data are expressed as mean ± s.e.m. The dotted line indicates the 50% MPE. Asterisks indicate significant differences compared to the vehicle (*p < 0.05, one-way ANOVA followed by Dunnett’s test).

**FIGURE 3:**
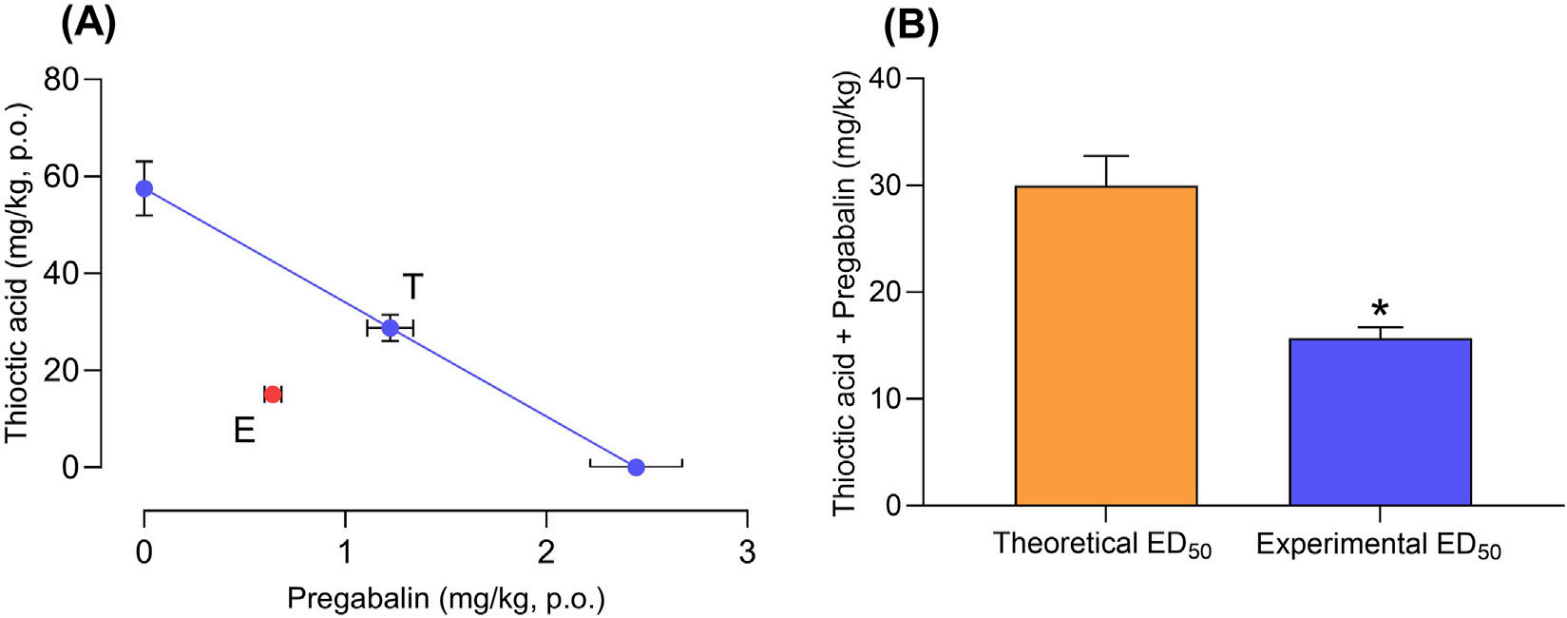
Synergistic interaction between pregabalin and thioctic acid in antinociceptive response. **(A)** Isobologram showing the theoretical (T) additive interaction between pregabalin (x-axis) and thioctic acid (y-axis) and the experimentally (E) observed combination effect in a neuropathic pain model. **(B)** Bar graph comparing theoretical and experimental ED_50_ values for the pregabalin–thioctic acid combination. The experimentally determined ED_50_ was significantly lower than the theoretical additive ED_50_, indicating a synergistic interaction. Data represent mean values (±s.e.m.); *p < 0.05 versus theoretical ED_50_ (t-test).

*Isobolographic analysis was based on ED_50_ values derived from individual dose–response curves (Figure 2) and 1:1 fixed-ratio combinations (Figure 3). Interaction index γ < 1 indicates synergy.

### 2.7 Statistical analysis

All data are presented as mean ± s.e.m. (n = 6 per group). Time-course data were analyzed using one-way ANOVA followed by Dunnett’s *post hoc* test for comparisons with the vehicle control. The area under the curve (AUC) was calculated using the trapezoidal method. Adverse effects such as sedation, motor impairment, or distress were qualitatively monitored; none were observed at combination doses, whereas mild sedation occurred at the highest pregabalin monotherapy dose. Differences were considered statistically significant at p ≤ 0.05.

## 3 Results

### 3.1 Pregabalin and thioctic acid produce dose-dependent antiallodynic effects in neuropathic rats

Oral administration of pregabalin (0.3–30 mg/kg) produced a robust, dose-dependent increase in mechanical withdrawal thresholds in rats subjected to L5–L6 spinal nerve ligation, which was interpreted as an antiallodynic effect. The most significant dose (30 mg/kg) restored thresholds to levels comparable to those of sham-operated controls, with peak effects observed between 2 and 4 h post-administration (Figure 1A). Similarly, thioctic acid (10–300 mg/kg) induced a graded antiallodynic response; however, its maximal efficacy matched that of pregabalin only at the most significant tested dose of 300 mg/kg (Figure 1B). When administered in a fixed 1:1 ratio based on ED_50_ equivalence, the pregabalin–thioctic acid combination (0.15–1.2 mg/kg + 3.6–28.8 mg/kg, respectively) yielded intermediate efficacy, with the 1.2 + 28.8 mg/kg dose producing the most sustained threshold elevation (Figure 1C). No overt adverse effects—such as sedation, motor impairment, or distress—were observed at combination doses, whereas mild sedation was occasionally noted at the highest pregabalin monotherapy dose.

### 3.2 Combination therapy displays synergistic interaction and reduced ED_50_

Dose–response analysis revealed that pregabalin achieved a %MPE of 108.2% ± 1.6% and an ED_50_ of 2.45 ± 0.23 mg/kg (Figure 2A), whereas thioctic acid reached a maximum %MPE of 91.3% ± 1.8% and an ED_50_ of 57.49 ± 5.59 mg/kg (Figure 2B). Notably, the fixed-dose pregabalin–thioctic acid combination produced a %MPE of 72.3% ± 4.8% and an ED_50_ of 15.7 ± 1.0 mg/kg (Figure 2C; Table 1). Although ED_50_ of combination was higher than that of pregabalin alone, it was significantly lower than the theoretical additive ED_50_ calculated from monotherapy data (30.0 ± 2.8 mg/kg). These findings indicate that the combination achieved a comparable level of antiallodynia to higher-dose monotherapy but at substantially lower individual drug exposures.

### 3.3 Isobolographic analysis confirms pharmacological synergy

Isobolographic assessment demonstrated that the experimental ED_50_ of the combination fell well below the theoretical additive line (Figure 3A). The calculated interaction index (γ) was 0.524 (95% CI: 0.41–0.66), indicating a synergistic effect. Statistical comparison of experimental versus theoretical ED_50_ values confirmed the significance of this deviation (p < 0.05, Student’s t-test; Figure 3B; Table 2). These findings support a synergistic interaction between pregabalin and thioctic acid when co-administered in equieffective proportions. This synergy is consistent with complementary mechanisms of action—central α_2_δ-1 subunit modulation by pregabalin and peripheral oxidative stress–mediated ion channel modulation by thioctic acid—providing convergent suppression of nociceptive transmission.

## 4 Discussion

Neuropathic pain remains a major therapeutic challenge, with current pharmacological treatments often yielding incomplete relief and being constrained by dose-limiting adverse effects (Kaye et al., 2025). In this context, rational polypharmacy—combining drugs with distinct mechanisms—has emerged as a strategy to enhance analgesic efficacy while minimizing side effects (Yorek, 2024). The present findings demonstrate that co-administration of pregabalin and thioctic acid produces a synergistic antiallodynic effect in the L5–L6 spinal nerve ligation model. Isobolographic analysis confirmed that the combination produced significantly greater efficacy than predicted by simple additivity, enabling a reduction in total dose without compromising therapeutic benefit (Figure 3; Table 2). Importantly, no overt sedation, motor impairment, or distress was observed at combination doses, whereas mild sedation occurred at the highest pregabalin monotherapy dose, suggesting a potential tolerability advantage.

Pregabalin, a ligand at the α_2_δ-1 subunit of voltage-gated calcium channels, is a mainstay in the treatment of neuropathic pain. Despite its efficacy, its clinical use is often limited by central nervous system side effects—such as somnolence, dizziness, and ataxia—particularly at higher doses (Zaccara et al., 2011). Thioctic acid, an endogenous antioxidant with known anti-inflammatory and metabolic regulatory properties, has demonstrated analgesic effects in both preclinical and clinical models, notably in diabetic neuropathy (Mijnhout et al., 2012; Papanas and Ziegler, 2014; Hsieh et al., 2023; Yazğan et al., 2023). Its mechanisms include the modulation of TRPV1 channel activity and expression through antioxidant effects, as well as the inhibition of the NF-κB signalling pathway. Additionally, it exhibits redox-sensitive inhibition of CaV3.2 T-type calcium channels, a molecular target that is increasingly implicated in the sensitization of nociception. These actions reduce neuronal excitability and alleviate nociceptive signalling (Lee et al., 2009; Zhang et al., 2020; Yazğan et al., 2023). Given pregabalin’s central α_2_δ subunit modulation and thioctic acid’s peripheral ion channel and oxidative stress–mediated effects, the observed synergy is pharmacologically plausible.

Although both agents are effective as monotherapy, the nature of their pharmacological interaction has remained poorly characterized. Previous rodent studies suggested an additive effect (Cruz-Alvarez et al., 2018), while clinical trials have yielded mixed results. Park et al. (2022) observed enhanced efficacy at low doses of pregabalin when combined with thioctic acid, whereas Gilron et al. (2024) found no clear benefit relative to monotherapy. Here, the combination achieved robust analgesia at a total dose nearly 50% lower than predicted for additivity, as indicated by an interaction index (γ) of 0.524 (95% CI: 0.41–0.66; Table 2). This pharmacodynamic synergy was evident in both the time-course of mechanical threshold restoration (Figure 1C) and the %MPE dose–response analysis (Figure 2C), and was supported by consistent ED_50_ values (15.7 ± 1.0 mg/kg) across replicated experiments (Table 1). These preclinical findings align with a broader body of evidence supporting mechanism-based drug combinations to reduce required doses and potentially improve safety profiles (Zamora-Diaz et al., 2025).

The experimental model employed—L5–L6 spinal nerve ligation—is a well-established paradigm that recapitulates key features of clinical neuropathic pain, including mechanical allodynia, and offers high predictive validity for therapeutic response (Kim and Chung, 1992; Kiso et al., 2008). Nonetheless, several limitations merit discussion.

First, while isobolographic analysis remains the reference standard for evaluating drug interactions, it does not delineate underlying mechanisms. The relative contributions of spinal α_2_δ channel inhibition, peripheral antioxidant action, or downstream convergence at nociceptive relay circuits remain unknown. Electrophysiological recordings, calcium imaging, and molecular analyses in dorsal root ganglia and spinal cord tissue may elucidate these interactions.

Second, the experiments were conducted exclusively in female rats. This choice was intentional to address the sex imbalance in preclinical synergy studies, most of which use male rodents (Mogil, 2020), but it necessarily limits generalisability. Given the known sex-dependent differences in pain processing, immune signalling, and drug metabolism, future studies should investigate whether this synergy also extends to male subjects. Likewise, testing in other models—including chemotherapy-induced or traumatic nerve injury—would strengthen generalizability.

Third, the compounds were administered orally to reflect clinical use, but potential pharmacokinetic interactions were not addressed. Thioctic acid may modulate intestinal absorption, first-pass metabolism, or blood–brain barrier transport of pregabalin. Comparative pharmacokinetic studies, including plasma and CNS levels under monotherapy and combination therapy, are needed to distinguish pharmacodynamic from disposition-based synergy.

Fourth, the present study focused on the acute antiallodynic effects up to 8 h post-dose. It remains unclear whether chronic co-administration would sustain the synergistic effect or be attenuated by tolerance, receptor desensitization, or compensatory neuroplasticity. Longitudinal dosing studies will be crucial in assessing the durability of the response and guiding the design of future clinical trials.

Finally, the isobolographic analysis was based on a single fixed 1:1 ED_50_ ratio. While this approach enables the quantification of rigorous interaction, it does not identify the optimal dose ratio for clinical translation. Future studies should explore alternative fixed ratios to define the most favourable balance between efficacy, safety, and tolerability. Exploring multiple fixed ratios could identify the most favourable balance between efficacy, tolerability, and patient adherence.

In summary, the current data provide robust preclinical evidence that the combination of pregabalin and thioctic acid yields synergistic antiallodynic effects in a rat model of neuropathic pain. This synergy enables effective pain control at lower doses than required for either monotherapy, potentially reducing adverse effects associated with higher single-agent dosing. These results support further evaluation in chronic dosing paradigms, inclusion of both sexes, and pharmacokinetic–pharmacodynamic studies to optimize translation. These findings support the further development of this combination as a mechanism-based, dose-efficient therapeutic strategy for neuropathic pain and justify continued evaluation in chronic models across both sexes and translational clinical studies.

## Data Availability

The raw data supporting the conclusions of this article will be made available by the authors, without undue reservation.
